# Patient Disease Perceptions and Coping Strategies for Arthritis in a Developing Nation: A Qualitative Study

**DOI:** 10.1186/1471-2474-12-228

**Published:** 2011-10-10

**Authors:** Nina N Niu, Aileen M Davis, Laura M Bogart, Thomas S Thornhill, Luis Alcantara Abreu, Roya Ghazinouri, Jeffrey N Katz

**Affiliations:** 1 Orthopedic and Arthritis Center for Outcomes Research, Brigham and Woman's Hospital, 75 Francis Street BC-4-4016, Boston, 02115, USA; 2 Division of Rheumatology, Immunology and Allergy, Brigham and Women's Hospital, 75 Francis Street, Boston, 02115, USA; 3 Department of Physical Therapy, Brigham and Women's Hospital, Tower 2C 75 Francis Street, Boston, 02115, USA; 4 Division of General Pediatrics, Children's Hospital Boston, 300 Longwood Avenue, Boston, 02115, USA; 5 Department of Pediatrics, Harvard Medical School, 25 Shattuck Street, Boston, 02115, USA; 6 Department of Orthopedic Surgery, Hospital General de la Plaza de la Salud, Avenida Ortega y Gasset, Dominican Republic; 7 Division of Healthcare and Outcomes Research, Toronto Western Research Institute, 399 Bathurst St. Toronto, Canada; 8 Arthritis Community Research & Evaluation Unit, Toronto Western Research Institute, 399 Bathurst St., Toronto, Canada; 9 Graduate Departments of Health Policy, Management and Evaluation and Rehabilitation Science, University of Toronto, 500 University Avenue, Toronto, Canada; 10 Department of Physical Therapy, University of Toronto, 500 University Avenue, Toronto, Canada

## Abstract

**Background:**

There is little prior research on the burden of arthritis in the developing world. We sought to document how patients with advanced arthritis living in the Dominican Republic are affected by and cope with their disease.

**Methods:**

We conducted semi-structured, one-to-one interviews with economically disadvantaged Dominican patients with advanced knee and/or hip arthritis in the Dominican Republic. The interviews, conducted in Spanish, followed a moderator's guide that included topics such as the patients' understanding of disease etiology, their support networks, and their coping mechanisms. The interviews were audiotaped, transcribed verbatim in Spanish, and systematically analyzed using content analysis. We assessed agreement in coding between two investigators.

**Results:**

18 patients were interviewed (mean age 60 years, median age 62 years, 72% women, 100% response rate). Patients invoked religious and environmental theories of disease etiology, stating that their illness had been caused by God's will or through contact with water. While all patients experienced pain and functional limitation, the social effects of arthritis were gender-specific: women noted interference with homemaking and churchgoing activities, while men experienced disruption with occupational roles. The coping strategies used by patients appeared to reflect their beliefs about disease causation and included prayer and avoidance of water.

**Conclusions:**

Patients' explanatory models of arthritis influenced the psychosocial effects of the disease and coping mechanisms used. Given the increasing reach of global health programs, understanding these culturally influenced perceptions of disease will be crucial in successfully treating chronic diseases in the developing world.

## Background

Arthritis is a leading cause of pain, functional limitation and disability worldwide [1-3]. Little is known about the burden of arthritis in developing-world settings. However, a substantial body of research has illuminated ways in which patients in *developed *countries are affected by arthritis. For example, osteoarthritis (OA) and rheumatoid arthritis (RA) often lead to physical limitations and depression [4-7]. Also, women with OA tend to report more severe pain and functional limitation as compared to men with similarly advanced disease [8]. Research on how patients in the developed world manage their arthritis has categorized coping strategies as active, involving attempts to manage or alter the course of the illness, and passive, characterized by disease avoidance and relinquishment of disease control [9]. Active coping strategies include participating in physical therapy, while passive coping includes withdrawing from social activities [10]. Active coping is typically associated with lower levels of pain and psychosocial distress than passive strategies [11,12]. Patients in developed countries use active coping strategies more frequently than passive strategies [13].

Given the increasing prevalence of arthritis worldwide [14,15] and the increasing reach of global health programs dedicated to alleviating the burden of chronic disease, research is needed to examine whether conclusions from research in developed countries also apply to developing countries. The objective of this study is to examine the illness experience of arthritis, including disease perceptions, physical and psychosocial effects of disease, and coping strategies of economically disadvantaged patients with advanced arthritis in the Dominican Republic, a society in which resources are scant, belief systems about disease differ from those of Western cultures, and gender roles are rigidly delineated [16].

We used semi-structured qualitative interviews to examine how patients' perceptions of the etiology of their arthritis [17] influence how they are affected by the disease and the strategies they use to cope with their illness. Our specific research questions included: How do patients conceptualize their disease and its etiology? To what extent does arthritis affect patients' abilities to continue fulfilling their social roles? Do informal and intangible resources - such as family, friends and religion - play prominent roles in managing arthritis in a setting in which professional care is difficult to obtain? And lastly, do men and women differ in the ways that they experience and cope with the disease? The overarching motivation for our work is that understanding how patients with limited resources in the Dominican Republic perceive, are affected by, and cope with their illness will help to improve treatment outcomes and the design of care delivery systems for individuals with arthritis in developing nations.

## Methods

### Setting

The Dominican Republic occupies the eastern 2/3 of the island Hispaniola, located between the Caribbean Sea and the North Atlantic Ocean. The population of the nation is approximately 9.8 million, with roughly 4.1 million citizens (42.2%) living below the international poverty line. The Dominican Republic is divided into 31 provinces and the National District. More than 60% of the population lives in urban areas and the population density is 197 persons per square kilometer. The adult literacy rate is approximately 85%. The economy is largely based on the service and agricultural sectors, yielding a per capita GDP of $8,300, or roughly 17.9% of that in the United States. In addition, marked financial inequalities exist: the poorest half of the population accounts for less than 20% of the national GDP, while the richest tenth possess more than 40% [18]. While all citizens are covered by a public healthcare system, only basic healthcare needs are included in this plan and advanced treatments such as total joint replacements are generally not accessible to the poor.

Significant advances in gender equality have occurred in the Dominican Republic over the past three decades. The organization *Mujeres en Desarollo Dominicana Inc *(Women for Dominican Development, MUDE), established in 1980, works to improve the quality of life for Dominican Women. Beginning in 1990, government parties started to incorporate women's issues into their political agendas. In 1997, Congress passed two important laws promoting women's private and public rights. The first, *La ley contra la violencia intrafamiliar *(Law against family violence) made all forms of domestic violence illegal and punishable by law. The second law established a quota system that required women to comprise 25% of city council candidates; this quota was increased to 33% in 2000. In 2000, the citizens of the Dominican Republic elected its first female president, Milagros Ortiz Bosch [19].

However, Dominican women continue to face gender-related challenges. Physical violence against women continues to occur, despite the 1997 law against domestic violence. According to a study by *Dominican Today *of 3,400 women, 44% have been victims of physical or non-physical abuse, typically at the hands of the women's husbands and boyfriends [20]. Women perform the majority of unpaid domestic work within families, limiting their freedom to seek employment opportunities outside of the household. Furthermore, the women who do earn salaries are more likely than men to be employed in the informal and services sectors, which are traditionally lower paid, less stable and have poorer working conditions. Thus, the culture of machismo, defined as a strong sense of masculine pride or exaggeration exertion of masculinity continues to exist and women remain more socially and economically vulnerable than men [21].

### Participants

The study was conducted with patients participating in OpWalk Boston, a philanthropic, not-for-profit, volunteer organization that provides pro bono total joint replacement (TJR) to patients with limited financial resources in the Dominican Republic. The OpWalk Boston team, consisting of about 50 individuals has established a partnership with Hospital General de la Plaza de Salud in Santo Domingo, Dominican Republic, and has been travelling to this hospital for the past 4 years to perform the surgeries. We operate on approximately 55-60 joints in ~ 45 patients annually, a number limited by the financial and human resources of the program and the size of the inpatient unit where patients are admitted.

Patients participating in OpWalk Boston learned of the program through advertisements on television, newspaper and radio, by word of mouth, or by physician referral. There was no formal test for financial eligibility. Patients were deemed eligible if they did not have health insurance that would cover the joint replacement and could not purchase the surgery out of pocket. To determine medical eligibility, patient radiographs and case histories were reviewed by Dominican and American surgeons. Patients were required to have symptomatic, radiographically advanced knee and/or hip arthritis. The first 50 patients who were both financially and medically eligible were selected into the program.

### Patient Recruitment

All 47 patients enrolled in OpWalk Boston 2010 were admitted to Hospital General de la Plaza de la Salud on March 18, 2010. Their surgeries occurred over the next few days. A bilingual investigator, who was a medical student from Boston working with the OpWalk Boston team approached patients during this pre-operative period and provided a written invitation to participate in the study. Patients were selected randomly to minimize bias. We chose to sample patients pre-operatively because we felt that their perspectives on arthritis might change in their post-operative state. Given time and personnel constraints, we were not able to approach all patients in OpWalk Boston for interview. We randomly approached 18 pre-operative patients to obtain our study sample of 18 individuals (100% response rate). Once a patient had surgery, he or she was no longer eligible for the study. We enrolled until there were no further patients remaining who had not yet undergone surgery. Candidates were given a Spanish consent form and consent was obtained from all participants.

### Procedures

One-to-one, semi-structured interviews were conducted in the participants' hospital rooms. Semi-structured interviews allow for focused, yet conversational communication and offer flexibility to elaborate on issues or raise new discussion topics [22].

The structure of the interview followed a moderator's guide (Table 1) developed through discussions with Dominican surgeons, clinicians and Dominicans immigrants living in the US. Broad topics included the patient's family and social support networks, the patient's understanding of disease etiology, and coping mechanisms used. The interviewer broached topics using broad, open-ended questions. For example, to understand the patient's explanatory model, the investigator asked "How do you think arthritis works?" To encourage further conversation on a particular topic, the interviewer asked more detailed questions. For example, follow-up questions regarding the patient's understanding of disease included "Why do you think you have arthritis?" The interviews were conducted in Spanish and lasted approximately 45 minutes. All were recorded on audiotape and transcribed verbatim in Spanish.

**Table 1:** Moderator's Guide for Interview

**Topics**	**Questions**
**Demographics**	Do you live in a rural or urban setting?
	What is your marital status?
	How many people are in your home?
	Do you have any other co-morbidities?
	Who do you spend time with throughout the day? Who do you go to if you are having trouble?
	
**Arthritis Story**	When did you first feel that you had "problem with your joints"?
	How did you feel physically?
	How did you feel mentally?
	
**Explanatory Model of Arthritis**	What do you know about arthritis (how do you think it works)?
	Where did you get your information about arthritis?
	Why do you think you have it?
	
**Effect of Arthritis on Life**	What is the most significant way that arthritis has affected your life?
	What are 3 activities that you can no longer do because of arthritis?
	What are 3 activities that you still can do with arthritis?
	How has arthritis affected your role as parent?
	How has arthritis affected your role as spouse?
	Has arthritis made you more happy or sad about life?
	
**Non-medical Resources**	Who has helped you at home (formal & informal)?
	Who has helped you in the community (formal & informal)?
	Who has helped you at work (formal & informal)?
	What has made it difficult to obtain support?
	What has made it easy to obtain support?
	When did you start asking for support?
	
**Medical Resources**	Have you seen a doctor?
	Have you seen any other types of healthcare providers?
	Have you tried any medications?
	Have you used any devices?
	Who taught you how to use them?
	What seems to work best?
	How much do you pay for office visits/medications/devices?
	What has made it difficult for you to obtain care?
	When did you seek care?
	
**Total Joint Replacement**	What do you know about the surgery you will receive?
	Who told you this?
	What do you expect from surgery? What is the chance that you'll have a complication like pneumonia or a heart problem? What is the chance that the surgery will take the pain away?
	What do you expect the recovery to be like? Do you expect that it will be painful, or that it will not be painful? How long do you think will it take until you are able to walk as much as you want?
	If you didn't qualify for OpWalk, what would you have done?
	
**Future Hopes**	What are your hopes for the future?
	How do you think your life will change once you have a new knee/hip?
	What are 2 or 3 activities that you look forward to being able to do after surgery?

### Analyses

Our analysis was based in grounded theory, which does not assume a pre-conceived theoretical perspective. Rather, grounded theory allows theories and hypotheses to arise from the data during the qualitative analysis process. In our research, patients were probed about broad topics related to their disease, and we formulated hypotheses regarding patients' belief systems after considering the narratives as a whole.

The transcripts were analyzed in Spanish by American, bilingual coders using content analysis, a method that allows the researcher to systematically classify large amounts of textual material into themes in order to make valid inferences without assuming cause and effect relationships between segments of text [23]. Three investigators read two randomly selected transcripts to gain a general sense of thematic content. The investigators then met to discuss themes and develop a preliminary coding method. We used open coding to create a scheme that consisted of three levels of abstraction [24]. Categories of words comprised the first level of abstraction. These categories corresponded to different portions of the moderator's guide and were formed through discussions among investigators. For example, one category under the section on explanatory models of arthritis was 'religion,' which included words such as 'church' and 'spirit.' Sentences containing these words were next read to determine context and subsequently to identify shared concrete themes among patients. For example, one theme in the category for religion included the belief that arthritis was a test from God. As similarities between themes were uncovered, they were grouped to form more abstract themes, such as patients' passivity toward the disease due to their belief that God controlled their fate.

One investigator then coded six more interviews, adding new themes and categories as they arose. After nine interviews were coded, three investigators met again to generate a final coding scheme. One investigator then re-coded all of the interviews using the finalized coding scheme. Coding agreement between two investigators, assessed for 198 themes in three interviews (66 themes per interview), was 92% (95% CI = 88% - 96%). The Cohen's Kappa coefficient for inter-rater agreement was 0.84 (95% CI = 0.76 - 0.92). The investigators reviewed the differences in coding and resolved them.

Throughout the analysis process, we remained aware that our Western conceptions of illness and disease could influence our interpretation of patients' narratives. To mitigate the effects of this potential bias on our results, we worked directly from the interview transcripts, rather than from our overall interpretations of patients' words and interviewer notes. In addition, we included a Dominican clinician as an investigator to ensure culturally appropriate and accurate objective interpretations of the text.

Quotations included in the manuscript text were translated into English.

Interviews were recorded on a tape recorder and were converted to mp3 files kept on a password-encrypted computer nightly. Patient identifiers were removed from interview transcripts, which are also kept on a password-encrypted computer system in the US. All study activities were approved by the institutional review boards at the Brigham and Women's Hospital and the Hospital General de la Plaza de la Salud.

## Results

Of the 47 patients participating in OpWalk Boston 2010, 18 were approached for an interview and all 18 consented. The remaining patients had their surgeries before they could be approached. Of the 18 patients interviewed, 13 (72%) were women. Six (33%) received total hip replacement (1 bilateral) and 12 (67%) received total knee replacement (2 bilateral). The mean age of the sample was 60 years (median 62 years, range 21 - 80 years). The gender, joint distribution and age of the 18 patients interviewed did not differ significantly from those who did not participate (72% women, 79% with knee replacement, mean age of 61 among non-participants). 15 patients did not complete secondary school education and 17 were unemployed. All patients followed the Christian religion; Catholicism was the most common denomination (n = 13), followed by Evangelism (n = 3) and the Church of 7^th ^Day Adventists (n = 2). Subjects had experienced symptomatic arthritis for a mean of 13 years prior to surgery (median = 17 years), with 11 patients reporting at least 10 years of symptoms.

### Overview of findings

From information gathered through over 20 hours of focused one-to-one interviews, we found significant connections among the 18 patients' beliefs in disease etiology, the impact of illness on their lives, and the coping strategies used to manage their disease. Patients generally invoked faith-based and environmental theories of disease causation including a belief that arthritis was God's will and that the disease arose from exposure to water earlier in their lives including bathing, spending time outdoors in the rain, and performing chores involving water such as washing dishes or clothes. Although all patients experienced pain and functional limitation due to arthritis, the nature of the disrupted social roles differed between women and men. The passive explanatory models appeared to influence the strategies used to mitigate the effects of their illness. These strategies frequently involved prayer and avoidance of chores involving contact with water. Patients also relied on family, church-based and neighborhood social support.

### Theme 1: Disease etiology

Over half of the patients (11 out of 18) believed that their arthritis had been instigated by contact with water. For example, when asked why she had arthritis, one patient responded: "Rainwater, from the sky. And the streets were full of water. I was young and I was in the water (age 68)." Other patients believed their arthritis originated from performing chores involving water. While men typically mentioned carrying water as part of their agricultural work, women spoke of household chores such as cleaning and washing: "Being with water a lot ... I was a fanatic about that ... cleaning and washing with a lot of water. Yes, I liked to always wash the floors, wash things, the house. I remember that my mother had said 'you're going to catch arthritis, that's harmful' (age 62). " Several individuals believed that using detergents during chores such as dishwashing and laundry contributed to their subsequent development of arthritis. Other subjects attributed arthritis to the water's cold temperature: "There are many people who suffer from the cold of the water (age 76)."

Half of the patients (9 out of 18) believed that God was responsible for their illness: "I think that God gave me the illness (age 63)." However, none of the patients believed that arthritis was a form of punishment from God. Rather, the patients felt that their arthritis was a test of their strength. According to one patient, "This problem is a test. A test. It's a test to see if I can continue fighting or not (age 21)."

When asked how they had come to believe in these etiologies, almost all patients stated that they had learned about their disease from friends and family, rather than from medical professionals. For example, one patient stated "this woman had arthritis. And I told her about my pain and she explained to me that it was arthritis (age 63)." In contrast, the patients reported that their physicians focused mostly on the fact that there was no cure for their illness: "they always told me that there was no cure, that there was no cure for arthritis (age 63)," and "they told me that there was no solution for me because it cost too much money (age 53)."

### Theme 2: Effect of Arthritis

#### Physical and social effects

While all patients reported severe physical limitations imposed by arthritis, most notably in walking, the social roles affected by these limitations were gender-specific. Women in the cohort described the intrusion of arthritis on their roles as homemakers and church members. Since the majority of patients believed that water had caused their arthritis, they avoided tasks involving water. For example, one woman cooked for her family, but no longer washed, stating, "There are things that I don't do. I don't wash. No liquid. I can cook. Wash no (age 64)." Other female patients took measures to prevent contact with water, such as putting on gloves before cleaning.

The church had an especially strong presence in women's lives as it afforded them leadership and organizational opportunities otherwise unattainable. Many of the female patients in OpWalk Boston were leaders of their prayer groups or church choirs. Arthritis interfered with the womens' church involvement because they were no longer able to walk to church. For those who were able to continue attending church services, many reported that they could not kneel to pray.

Male patients, on the other hand, appeared more concerned about their disrupted roles as wage earners and providers. Arthritis forced all of the men interviewed to leave their paid jobs because according to one of the male patients, "The worst is that if you can't walk, you can't work." One of the males patients felt that his life had been delayed because he could no longer work ("Yes, I have been significantly set back because I can neither walk nor work (age 63)") while another reported feeling sad ("When this disease began to enter my body, I felt sad because I could not work (age 66).") Three of the five men reported that arthritis disrupted relationships with women. One of these patients reported: "Sometimes she wants to do something that I can't do ...or she wants to go out and I can't because of the pain (age 51)." According to another male patient, his disease and lack of occupation had caused his wife's personality to change: "she has changed a lot since I've been like this, sick and not working (age 48)." Another of the single male patients stated that his arthritis had lead him to become unemployed and thereby unable to find a girlfriend; when asked if it was his arthritis specifically that created this difficulty, he responded, "Yes, it's very hard without a job. You have to work to be able to do that (age 62)."

#### Psychological effects

Most patients reported feeling sad or depressed due to the constant pain and physical limitations imposed by the disease: "I was sad, you might guess, because pain is not easy (age 54).*" *In a society where women were traditionally homemakers, many women expressed sadness in losing the ability to adequately cook and clean. For male patients, the inability to earn a salary and loss of strength threatened their sense of masculinity. As one man stated, "I was a man of vigor, a man of labor, a strong man. When this disease began...I lost my strength. I couldn't lift 40 liters of water (age 66)."

### Theme 3: Coping

To manage their disease, patients used a combination of conventional and alternative coping methods that included pharmacologic, social, and faith-based strategies. Whereas all patients sought financial support from family and friends, women seemed to be more successful than men in mobilizing social support networks to cope with the emotional, physical and social consequences of their arthritis.

#### Pain treatments

Since patients' beliefs about disease etiology were mostly faith-based or environmental, they took few measures to prevent functional impairment from their disease, instead focusing on immediate symptom relief. Thus, the most frequently used treatment was pain medication. Almost all reported using diclofenac, a nonsteroidal anti-inflammatory drug (NSAID). Several patients also rubbed menthol creams ('mentoles') on their joints to alleviate pain. Citing their belief that water had caused their arthritis, the patients attached great importance to drainage of knee effusions: "I need to go to the doctor to get the liquid removed (age 63)."

#### Financial support

All patients sought financial support for medical care from their family, friends and neighbors. Most patients collected small amounts of money from multiple individuals by asking their children, circling the neighborhood, or calling upon their friends from church. Despite this financial support system, all patients reported difficulty paying for their doctor's visits and as a result, cut down on seeking care: "Sometimes I want to go, but I have to wait. It makes it difficult at times (age 80)." Another patient noted: "After, I stopped seeing him because of the economy. There was not a lot of money in my family (age 21)." Several patients explained that had they not been accepted into the program, they would have died in pain from their disease: "I don't have money for the operation. [Without this program,] I would remain like this, die like this (age 63)" and "There would be no solution, [this operation] costs too much money, I'm poor, what could I do? I didn't have anything (age 53)."

#### Social support: help to continue fulfilling social roles

Patients mobilized extensive support systems consisting of family, friends and neighbors to assist them through the day-to-day tasks made difficult by arthritis. These support networks differed by gender.

For male patients, the spouse or children typically provided physical support. None of the male patients mentioned seeking emotional support from family or friends. One male patient, who in the past, stated that he had friends and a girlfriend, had even withdrawn from all social relationships since the onset of his disease. When asked if he had friends currently, he responded: "Friends? No. Acquaintances yes, but no friends (age 51)."

Female patients appeared to rely on a stronger social support network of friends and neighbors to help them cope with emotional and physical effects of their illness. For example, one patient said of her friends: "They help me a great deal ... you know, things that motivate you to continue on, to fight (age 21)." Another patient's neighbor helped her with chores: "They help me to do whatever chore in the house (age 67)." Many of the women also received tangible and emotional support from other female members of their church. In addition, one younger female patient belonged to a Dominican organization for women living with disabilities titled *Círculo de mujeres con discapacidades *(Circle for Women with Disabilities). Notably, when asked about who helped them perform the household tasks that they could no longer do, none of the women mentioned their husbands. Rather, they relied on other female relatives, friends, and neighbors. For example, one woman responded: "My daughter. She is the one who helps me. And my grandaughter. The two of them [help me with] the chores at home. I cook, but they clean the house (age 63)."

#### Religious coping: to cure the disease

Since many patients believed that their arthritis represented God's way of testing their faith, they trusted that demonstrating their faith would ultimately convince God to cure their illness.

Almost all of the patients prayed. One patient said: "I prayed to God to cure me, to help me with everything, to reach out to me (age 70)." Consistent with their strong social affiliations with their church, several of the female patients reported benefiting from the prayer of others: "I attend a prayer group that helps me a lot too...they pray a lot for my sickness. The prayers help me a lot (age 67)." Thus the patients believed that through prayer, God would alleviate the symptoms of their illness. Furthermore, many patients believed that God had the power to cure arthritis: "I have a God who has the last word... I know that God cures (age 67)."

At the same time, patients also described forming partnerships with God, in which the patients actively prayed to God, in order to acquire the strength to endure their disease. According to one patient, "God has helped me a lot ... I have a lot of faith. He gives strength to people, patience (age 53)." Other patients stated, "God has helped me to learn to live, to deal with the pain (age 54)" and "I put everything in the hands of God. I have always been with Him...through the bad and the good (age 68)." Furthermore, they believed that their prayers convinced God to support them in ways that would not have occurred had they not prayed: "I pray. I put myself in his hands. I believe in him. And he helps me (age 80)," and "My bones don't hurt me so much because I started [praying] with this prayer group (age 67)." These individuals felt empowered from their prayers to God believing that through these prayers, they could gain a sense of control over their illness.

For many of these patients, God's acknowledgement of their struggles and faith came in the form of acceptance into the OpWalk Boston program. According to one patient, God had empowered the OpWalk Boston team to carry out God's will: "God is going to use them and they are going to do their job they way God wanted them to ... [with] wisdom, intelligence, that God has given to the doctors (age 66)." Thus patients turned to God and religion to manage and even cure their arthritis, attributing both their ability to endure their disease and the eventual receipt of their operation to their partnership with God.

## Discussion

This is the first qualitative study we are aware of that examines the burden of arthritis in a developing nation. We found that patients' conceptions of disease etiology influenced the psychosocial effects of arthritis and the mechanisms they used to cope with their illness. Specifically, patients invoked environmental and faith-based theories of arthritis causation, believing that their disease had been caused by contact with water, God's will, or a combination of both. These explanations appeared to allow the patients to accept their illness. The patients' belief that God would one day rid them of their arthritis, combined with the relative inaccessibility of pharmacological and surgical interventions, influenced them to use religious coping mechanisms, such as praying and forming partnerships with God.

We note several gender differences in the social consequences of arthritis and the networks available to help patients endure their illness. While female patients felt that they could no longer continue fulfilling their roles as homemakers and church members, men reported difficulties in functioning as workers and breadwinners. Male patients relied primarily on physical support from family members. In contrast, female patients mobilized a more extensive social network of family, friends and neighbors to help them cope with the physical and emotional consequences of their disease, allowing them to better maintain their social roles.

Our findings add to prior literature on the worldwide burden of chronic disease, specifically regarding conceptions of pain and disease etiology, the role of religious coping, and the importance of social support.

For example, a study of Canadian adults found that individuals with arthritis assigned different meanings to symptoms such as pain, stiffness and fatigue as compared to those without disease. In addition, patients with arthritis typically normalized their disease, viewing arthritis as an expected part of aging [25]. In contrast, many patients in our study attributed their disease to God and environmental origins. Similar to our findings, a qualitative study of 28 Dominican women with lymphatic filiarisis, a mosquito-transmitted parasitic disease, found that women attributed their condition to diverse cause such ranging from pregnancy, to varicose veins, to supernatural causes such as having small birds reside in their leg [26].

The use of religious coping has been researched extensively. For example, a study consisting of 6,082 face-to-face interviews with healthy, community-dwelling US citizens revealed that African American and Black Caribbean patients who were married or female were more likely to use religious coping as compared to male or single patients. Higher levels of education were associated with lower utilization of prayer during stressful situations such as disease [27]. From our small sample of 18 Dominican patients, we did not observe gender or age disparities in the use of religious coping. However, women appeared to often rely on religious groups, such as church choirs, while the men mostly engaged in individual religious activities, such as praying. Another study, in which 35 patients with rheumatoid arthritis were asked to keep a daily diary of their disease experience for 30 consecutive days, found that individuals who reported daily spiritual experiences, such as praying, had higher levels of positive mood and greater tolerance of pain [28]. A study involving interviews of 109 Latinos (89.5% female) living in the US with musculoskeletal disease found that 38.1% of patients used religion to help them cope with their illness [29]. Similar to the patients in our cohort, these women stated that their faith in God helped them to endure their disease and that their religion provided them with source of comfort and hope. Furthermore, in 2008, Kirmayer et al. described how religion can imbue transcendent meaning to the pain experience. In our study, patients' perception that they were 'chosen' by God to endure the pain of arthritis and ultimately overcome the disease points to a larger role for religion in the explanatory model of arthritis than simply a method of understanding disease etiology and coping with daily pain and functional limitation. The perception of being 'chosen' by God interweaves the arthritis experience with spiritual and ritual realms that influences how patients' of pain and suffering are constructed, articulated and sustained [30].

Other data have shown the importance of social support in Hispanic patients with chronic disease. A study of 98 Latina women in the US with arthritis found that having emotional support for their illness predicted greater psychological well-being and less psychological distress [31]. In our study, almost all patients relied strongly upon support from family, friends and/or neighbors for both social support and to help them continue fulfilling their social roles.

Several limitations should be noted. Our sample size was limited by the finite number of OpWalk Boston patients and the time constraints of the interviewer. In addition, participants of OpWalk Boston represent a select sample of individuals who successfully sought surgical care for their arthritis. While the interviews show that that these patients possessed a passive view of their illness, as shown in their beliefs in disease etiology and coping mechanisms, these data may actually underestimate the passivity of the Dominican population with arthritis as a whole. The vast majority of individuals with arthritis in the country did not apply to programs such as OpWalk Boston. Therefore, we might have found more passivity among patients with arthritis if we had chosen a different sampling strategy.

Furthermore, the researchers were members of a philanthropic care organization. It is conceivable that study participants were cautious about what they said to the researchers, leading to a reporting bias. In addition, bias in interpretation threatens qualitative studies such as ours. We minimized such biases by working from verbatim text during coding, evaluating consistency in coding and conducting iterative discussions of interview content with the research team. Finally, we acknowledge that our transcription and translation process may miss subtle aspects of language and culture. These would become more apparent in prolonged in-person conversations that permit interpretation of facial expressions and nuanced turns of phrase.

## Conclusion

In this qualitative study, we found that patients' conceptions of the etiology of their arthritis, which included beliefs that their disease had been caused by contact with water or God's will, appeared to allow them to accept their illness and influenced them to use religious coping mechanisms such as prayer. Our work has important implications for the care of patients with arthritis in developing nations and in the United States. Implementing arthritis treatment guidelines derived in developed countries for patients in developing nations may be ineffective if they are not introduced in the context of the patients' belief systems and with an understanding of their limited resources. Such plans may combine methods that are culturally congruent, inexpensive and safe -- such as prayer -- with proven interventions such as stretching and muscle strengthening. Consideration of culturally influenced disease perceptions is pertinent not only to international programs such as OpWalk Boston, but also to physicians caring for minority groups in the United States.

Our study is important in increasing sensitivity for cultural variations in perceptions in disease etiology and coping strategies. Future research could incorporate data from a larger group of patients with suitable comparison groups to make quantitative comparisons. Further studies performed on non-hospitalized Dominican patients with arthritis would also be helpful in understanding the burden of arthritis in the country. Understanding the distinctive impact of arthritis in a developing nation will provide insight to improving the care of patients with chronic conditions, which have become more prevalent as populations age across diverse global settings.

## List of abbreviations

OA: Osteoarthritis; RA: Rheumatoid Arthritis; OpWalk: Operation Walk; TJR: Total Joint Replacement; NSAID: Non-steroidal Anti-Inflammatory Drug.

## Competing interests

The authors declare that they have no competing interests.

## Authors' contributions

All authors read and approved the final manuscript. NNN, LMB, TST, LAA, RG and JNK contributed to study conception and design. NNN had the primary role of authoring the manuscript and data acquisition. NNN, JNK, AMB and LMB analyzed and interpreted the data. NNN, JEC, TST, LAA, RG, KMO, and JNK drafted and revised the manuscript.

## Pre-publication history

The pre-publication history for this paper can be accessed here:

http://www.biomedcentral.com/1471-2474/12/228/prepub
